# IL-7 treatment augments and prolongs sepsis-induced expansion of IL-10-producing B lymphocytes and myeloid-derived suppressor cells

**DOI:** 10.1371/journal.pone.0192304

**Published:** 2018-02-21

**Authors:** Upasana Kulkarni, Christoph Herrmenau, Stephanie J. Win, Michael Bauer, Thomas Kamradt

**Affiliations:** 1 Institute of Immunology, Jena University Hospital, Jena, Germany; 2 Department of Anesthesiology and Intensive Care Medicine, Jena University Hospital, Jena, Germany; 3 Center for Sepsis Control & Care, Jena University Hospital, Jena, Germany; University Medical Center of the Johannes Gutenberg University of Mainz, GERMANY

## Abstract

Immunological dysregulation in sepsis is associated with often lethal secondary infections. Loss of effector cells and an expansion of immunoregulatory cell populations both contribute to sepsis-induced immunosuppression. The extent and duration of this immunosuppression are unknown. Interleukin 7 (IL-7) is important for the maintenance of lymphocytes and can accelerate the reconstitution of effector lymphocytes in sepsis. How IL-7 influences immunosuppressive cell populations is unknown. We have used the mouse model of peritoneal contamination and infection (PCI) to investigate the expansion of immunoregulatory cells as long-term sequelae of sepsis with or without IL-7 treatment. We analysed the frequencies and numbers of regulatory T cells (Tregs), double negative T cells, IL-10 producing B cells and myeloid-derived suppressor cells (MDSCs) for 3.5 months after sepsis induction. Sepsis induced an increase in IL-10^+^ B cells, which was enhanced and prolonged by IL-7 treatment. An increased frequency of MDSCs in the spleen was still detectable 3.5 months after sepsis induction and this was more pronounced in IL-7-treated mice. MDSCs from septic mice were more potent at suppressing T cell proliferation than MDSCs from control mice. Our data reveal that sepsis induces a long lasting increase in IL-10^+^ B cells and MDSCs. Late-onset IL-7 treatment augments this increase, which should be relevant for clinical interventions.

## Introduction

Sepsis syndrome is a life-threatening organ dysfunction resulting from a dysregulated host response to an infection [1]. Immunological dysregulation is one of the major pathological events in sepsis [2–4]. Over-exuberant inflammatory responses and immunosuppression can occur simultaneously [4–6]. Treatment strategies for sepsis have improved, consequently more patients survive the acute sepsis episode [4,7]. These patients are burdened with significantly increased morbidity and mortality as long-term sequelae of sepsis [8–10] and mortality is further increased in sepsis survivors who had a secondary infection [11]. Currently neither the extent nor the duration of sepsis-induced immunosuppression is known. Furthermore, it has been impossible to rule out pre-existing immunodeficiency in sepsis patients. While the factors responsible for this increased morbidity and mortality are still unknown it is likely that long-term survival of sepsis patients depends on overcoming sepsis-induced immunosuppression. It is currently unknown if all sepsis survivors eventually recover from immunosuppression or if immune-recuperation proceeds with different kinetics and outcomes or selectively for particular cell populations in different subjects.

Considering the failure of various clinical trials aimed at targeting the hyper-inflammatory mediators, particularly cytokines such as tumor necrosis factor-α [3,4,12], it is necessary to analyse the long-term immune-perturbations in sepsis [13,14]. Moreover, immunological alterations in sepsis survivors are prognostically relevant [5,6]. Thus, it is necessary to perform basic and translational studies to understand post-sepsis immune-regulation. Immunoregulatory cells, including regulatory T cells (Tregs), IL-10 producing B cells, myeloid derived suppressor cells (MDSCs) and double negative (DN) T cells are important to dampen immune responses and to prevent autoimmunity and allergy [15–20]. In contrast, the role of these cells in the long-term sequelae of sepsis is unknown. To study the immunological sequelae of sepsis, we used the model of peritoneal contamination and infection (PCI) [21]. In this model, approximately 50% of the mice survive the acute phase of sepsis. Therefore, it is possible to determine the magnitude, duration and long-term consequences of sepsis-induced changes in the frequency and function of immunoregulatory cell populations in this model. We analysed Tregs, IL-10 producing B cells, MDSCs and double negative (DN) T cells 1 week, 1 month and 3.5 months after sepsis induction, replicating the post-acute, late and very late time points, respectively. IL-7 is required for lymphocyte development and maintenance [22] and sepsis induces ablation of IL-7-producing osteoblasts [23]. Early IL-7 treatment has been shown to be a promising approach in a mouse sepsis model [24] and *ex vivo* studies with IL-7-treated lymphocytes from sepsis patients showed significant improvement in their function [25]. To determine the effect of IL-7 treatment on the immunophenotype of sepsis-survivors we also analysed the effects of late-onset IL-7 treatment on the immunoregulatory cell populations.

## Methods

### Mice

C57BL/6 mice were bred and maintained at the animal facility of the University Hospital Jena. All animal experiments were approved by the appropriate governmental authority (Thüringer Landesamt für Lebensmittelsicherheit und Verbraucherschutz; Registered Number 02–007/14) and conducted in accordance with institutional and state guidelines.

### Sepsis induction and IL-7 treatment

Sepsis induction in mice was performed as previously described [21]. Briefly, human stool samples were collected and stored at -80°C. Animals were randomly allocated to the sepsis or sham group. Sepsis was induced by intraperitoneal (i.p.) injection of 1.75 ml/kg body weight stool suspension, diluted (1:4) in saline. Sham mice received the equivalent volume of saline (i.p.). The septic mice received antibiotic treatment (meropenem 12 mg/kg, administered subcutaneously). The first antibiotic injection was performed 7 h post sepsis induction, after which it was given every 12 h for the next 3 days. Mice were monitored for symptoms including conjunctivitis, diarrhea, weakness and lack of movement. On average 50% of the mice died during the acute phase of sepsis (days 1–5). Surviving mice were used for the analysis of long-term sequelae following sepsis. The experimental scheme is depicted in S1A Fig.

From day 5–9 septic mice were either subcutaneously injected with PBS or recombinant human IL-7 (R&D Systems, 2.5 μg/mouse/day). Human IL-7 can bind and signal via the murine IL-7 receptor [26]. In order to stabilize the cytokine, IL-7 was mixed with a ten-fold higher concentration of an anti-human IL-7 antibody (clone M25; BioXCell) [27,28].

### Flow cytometry

After blockade of Fc receptors with anti-CD16/CD32 (clone 2.4G2, in house production), single cell suspensions were incubated for 15 min with conjugated antibodies against cell surface markers. For intracellular cytokine staining of T and B cells, cells were first incubated in RPMI 1640 medium with PMA (50 ng/ml, final concentration), ionomycin (500 ng/ml, final concentration), LPS (10 μg/ml, final concentration), and monensin (2 mM, final concentration) for 5 h in 48-well flat-bottom plates. After 5 h culture, the surface markers were first stained followed by fixation and permeabilization using BD Cytofix/Cytoperm and intracellular staining. Samples were analysed using a LSRII (BD Biosciences). Data were analysed using FlowJo software (TreeStar Inc.).

### Antibodies

The following anti-mouse antibodies and conjugates were used in the flow cytometry experiments: *Alexa Fluor 647*: CD19 (clone 1D3; eBioscience); *Alexa Fluor 700*: GR-1 (RB6-8C5, eBioscience); *APC*: CD11b (clone M1/70; eBioscience); IL-10 (JES5-16E3, eBioscience); *APC-eFluor780*: CD4 (clone GK1.5; eBioscience), CD8 (53–6.7, eBioscience); *eFluor-450*: Foxp3 (FJK16s, eBioscience); *FITC*: CD5 (53–7.3, eBioscience); *Pacific Blue*: CD1d (1B1, Biolegend), CD3 (clone 145-2C11, in house production); *PE*: IFN-γ (XMG-1.2, eBioscience); *PECy5*: γδTCR (GL3, eBioscience); *PECy7*: CD25 (PC61.5, eBioscience), NK1.1 (clone PK136; eBioscience).

### MDSC-T Cell co-culture assay

CD4^+^ T cells and Gr1^+^ MDSCs were isolated from total spleen cells using biotin-labeled antibodies followed by automatic MACS (autoMACS Pro Separator, Miltenyi Biotec). The CD4^+^ T cells were first labeled with eFluor 670-labeled cell proliferation dye (eBioscience) followed by co-incubation of 2 x 10^5^ cells with Gr1^+^ cells in the ratio 1:1 in 96 well round bottom plates. T cells were stimulated with anti-CD3/CD28 (3 μg/ml) antibodies. Proliferation of CD4^+^ cells was analysed 3 days later by flow cytometry.

### Statistics

Statistical calculations were performed using GraphPad Prism. Experiments with two groups were analysed by unpaired two-sided Student’s *t* test. Comparisons involving multiple groups were analysed in a two-stage procedure by one-way ANOVA. If the ANOVA indicated a significant difference between the groups (*P* < 0.05), all groups were further compared pairwise by Tukey's multiple comparison test. In case of comparisons involving multiple groups with non-parametric data, a Kruskal-Wallis test was performed. * *P* < 0.05, ** *P* < 0.01, *** *P* < 0.001. Data are expressed as mean ± SEM as indicated in the figure legends.

## Results

### Sepsis induces a sustained increase of IL-10^+^ B cells

The aim of this study was to evaluate the numbers and frequencies of immunoregulatory cell populations for 3.5 months after sepsis induction in the presence or absence of early IL-7 treatment. As expected in the PCI model [21], the mortality within the first five days after sepsis induction was > 40%. On day five, mice were randomly allocated to the IL-7 treatment group, which were treated subcutaneously with 2.5 μg recombinant human IL-7 daily from day 5–9, or the control group, which received no further treatment. Mortality was similar in both groups throughout observation period of 3.5 months (S1B Fig).

**Fig 1:**
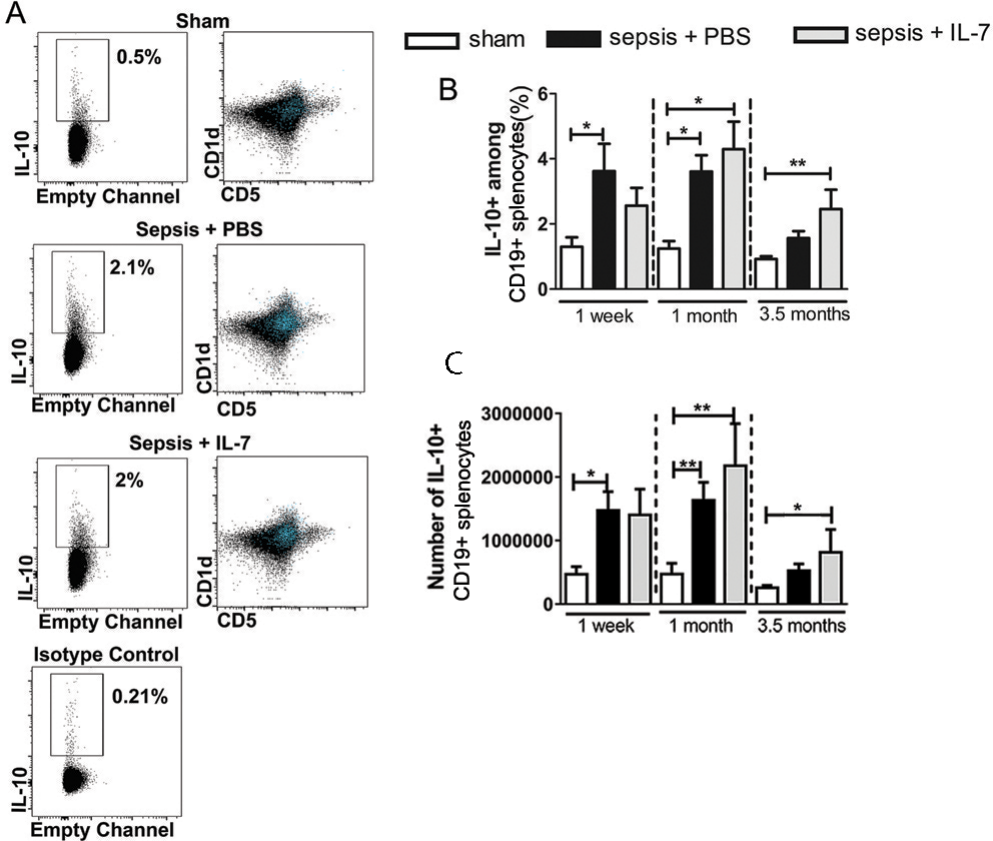
Sepsis induces a sustained increase of IL-10^+^ B cells. Mice were injected with PBS i.p. (Sham) or subjected to sepsis induction. IL-7 (Sepsis + IL-7) or PBS (Sepsis + PBS) was injected daily for 5 days from day 5–9 post sepsis induction. IL-10^+^ and CD1d^hi^ B cells from the spleen were analysed 1 week, 1 month and 3.5 months after sepsis induction. **(A)** Representative flow cytometry images from analyses 1 week after sepsis induction showing IL-10^+^ cells among CD19^+^ B cells (left panel) and distribution of IL-10^+^ cells (blue) with respect to CD1d and CD5 markers (right panel). **(B)** Frequency of IL-10^+^ cells among CD19^+^ B cells. (C) Number of IL-10^+^ CD19^+^ cells in the spleen. n (number of mice per group) = 6–10 (1 week), 9–16 (1 month), 7–16 (3.5 months). *P< 0.05, **P< 0.01 (ANOVA). Data are expressed as mean ± SEM. Data are representative of three experiments.

To examine if increased numbers of IL-10 producing B cells are a long-term outcome of sepsis, we performed IL-10 staining in CD19^+^ B cells from the spleens of septic and control mice (Fig 1A). IL-10 producing B cells have also been dubbed “regulatory B cells” (Bregs) and CD1d and CD5 are commonly used as surface markers for these IL-10 producing regulatory B cells [29]. Most of the IL-10^+^ B cells were in the CD1d^hi^/CD5^+^ population (Fig 1A). One week and one month after sepsis induction, both the percentage and the numbers of IL-10^+^ cells among B cells were increased threefold in mice with sepsis compared with non-septic (sham) mice (Fig 1B and 1C). 3.5 months after sepsis, IL-10 expressing B cells were still slightly, but no longer significantly, increased in septic mice. Interestingly, 1 month post-sepsis both the frequency and the numbers of IL-10^+^ B cells were higher in IL-7-treated septic mice than in sham mice and remained elevated even 3.5 months after sepsis induction (Fig 1B and 1C). IL-7 is essential for the development of B cells [30] and an increase in total B cells was also seen in the IL-7 treated mice, 1 month and 3.5 months after sepsis induction (S2 Fig). The results indicate that short-term treatment with IL-7 can result in long-term changes in the development of B cells including IL-10 expressing B cells.

### Sepsis does not induce a lasting expansion of Tregs

**Fig 2:**
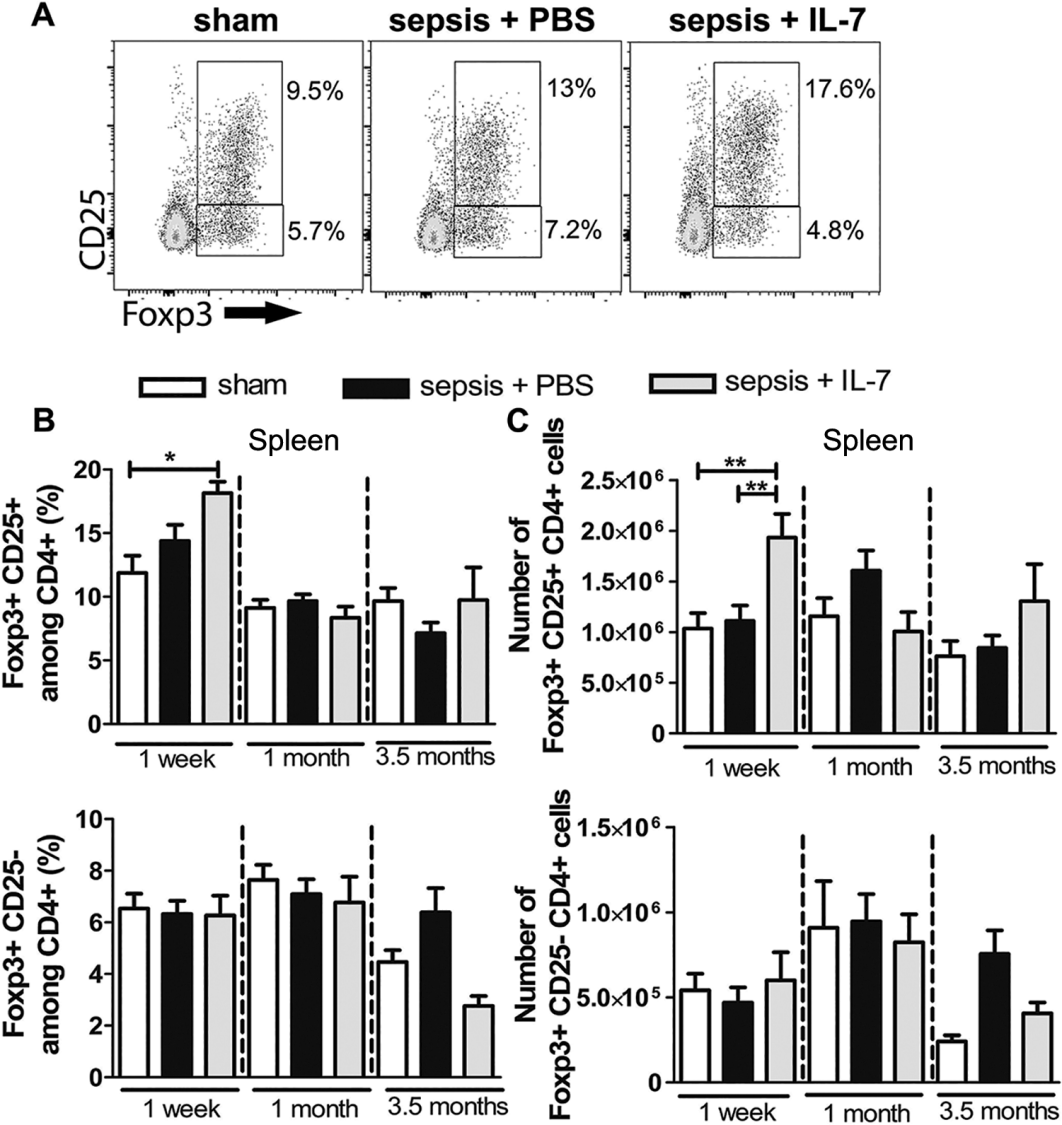
Sepsis induces a transient expansion of Tregs. Mice were injected with PBS i.p. (Sham) or subjected to sepsis induction. IL-7 (Sepsis + IL-7) or PBS (Sepsis + PBS) was injected daily for 5 days from day 5–9 post sepsis induction. CD4^+^ T cells from the spleen were analysed for expression of Foxp3 and CD25, 1 week, 1 month and 3.5 months after sepsis induction. **(A)** Representative flow cytometry images from analysis after 1 month showing CD25^+^ and Foxp3^+^ cells among CD4^+^ T cells. **(B)** Frequency of Foxp3^+^CD25^+^ (top) and Foxp3^+^CD25^-^ (bottom) cells among CD4^+^ T cells. **(C)** Number of Foxp3^+^CD25^+^CD4^+^ cells (top) and Foxp3^+^CD25^-^CD4^+^ T cells (bottom). n = 7–13 (1 week), 9–16 (1 month), 5–18 (3.5 months). *P< 0.05, **P< 0.01 (ANOVA). Data are expressed as mean ± SEM. Data are representative of three experiments.

Increased Treg frequencies in early sepsis have been reported in clinical and experimental studies [2,14,31–34]. To determine if increased Treg numbers are maintained as a long-term consequence of sepsis, we analysed the frequency and numbers of Tregs in spleen from septic and control mice at different time points after sepsis induction. Two distinct populations of Foxp3^+^CD4^+^ T cells were detectable: Foxp3^+^CD25^+^ and Foxp3^+^CD25^-^ (Fig 2A). Numbers and frequencies of both these populations were similar in the sepsis and control groups from 1 week until 3.5 months after sepsis induction. Interestingly, IL-7 treatment resulted in an immediate increase in the classical Foxp3^+^CD25^+^ Treg population, 1 week after sepsis induction (Fig 2B and 2C). In contrast, no increase in total CD4^+^ T cells was observed 1 week post sepsis induction in IL-7-treated septic mice (S3 Fig), indicating a preferential expansion of Foxp3^+^CD25^+^ Tregs upon IL-7 treatment. The increase in the Treg population following IL-7 treatment was only short-term and there were no significant differences in the frequency or numbers of Tregs between IL-7-treated mice and controls at 1 month and 3.5 months after sepsis induction (Fig 2B and 2C).

### Sepsis results in sustained activation of CD3^+^CD4^-^CD8^-^ T cells

**Fig 3:**
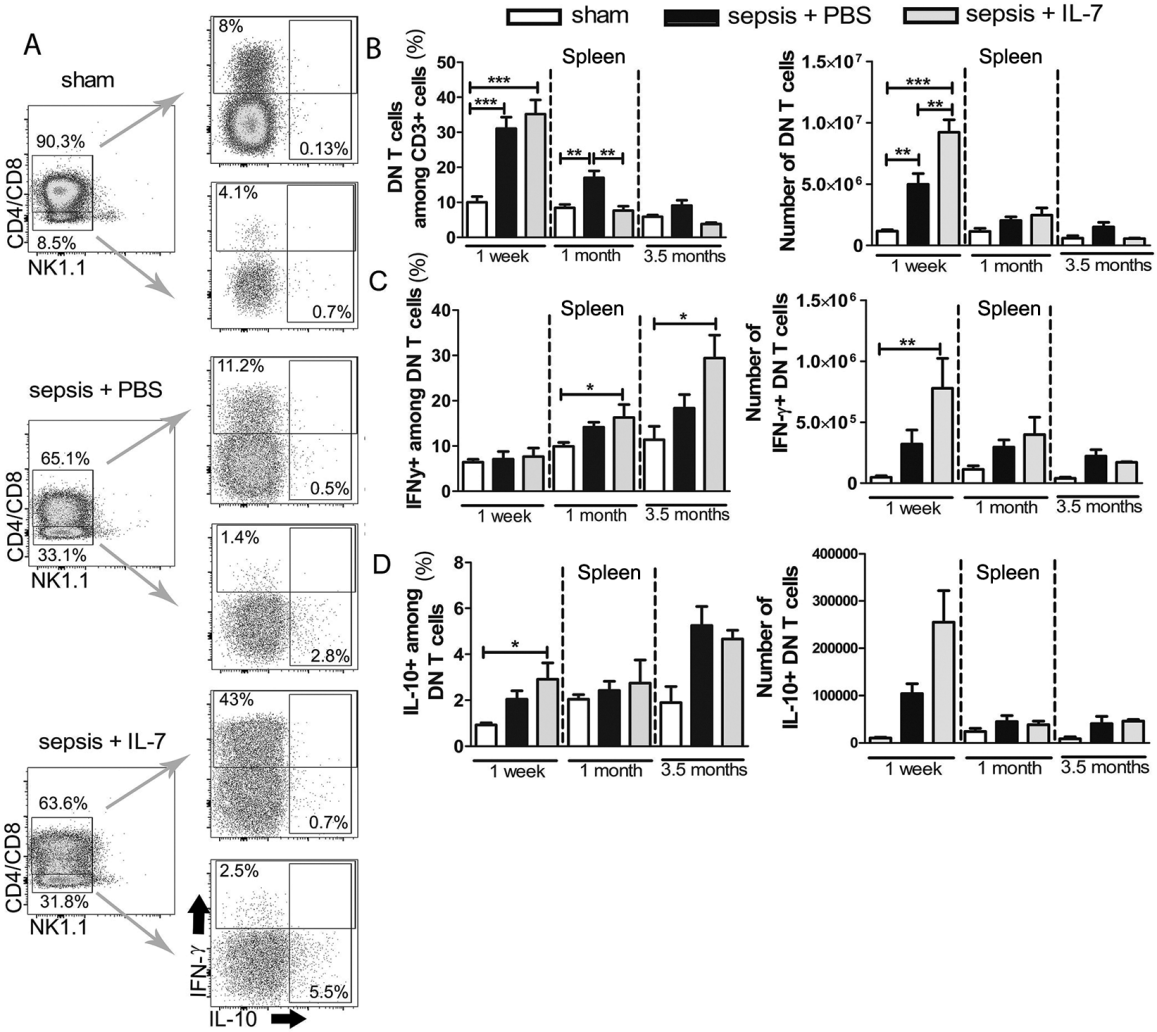
Sepsis results in sustained activation of DN T cells. Mice were injected with PBS i.p. (Sham) or subjected to sepsis induction. IL-7 (Sepsis + IL-7) or PBS (Sepsis + PBS) was injected daily for 5 days from day 5–9 post sepsis induction. The cytokine expression in CD3^+^NK1.1^-^γδTCR^-^CD4^-^CD8^-^ (double negative, DN) T cells from the spleen was analysed 1 week, 1 month and 3.5 months after sepsis induction. **(A)** Representative flow cytometry images from analysis after 1 week showing DN T cells and IFN-γ and IL-10 expression. **(B)** Frequency of DN T cells among CD3^+^ cells (left) and their absolute numbers (right). **(C)** Frequency of IFN-γ^+^ cells among DN T cells (left) and their absolute numbers (right). **(D)** Frequency of IL-10^+^ cells among DN T cells (left) and their absolute numbers (right). n = 7–13 (1 week), 5–10 (1 month), 3–6 (3.5 months) for IL-10 staining and 3–12 (3.5 months) for IFN-γ staining. *P< 0.05, **P< 0.01, ***P< 0.001 (ANOVA). Data are expressed as mean ± SEM. Data are representative of three experiments for the sham and sepsis + PBS groups. Data are representative of two experiments for the sepsis + IL-7 group.

CD3^+^CD4^-^CD8^-^ double negative T cells (DN T cells) that are neither NK T cells nor γδ T cells are believed to be T cells that escape negative selection in the thymus [20]. Some studies suggest that these cells play a pathogenic role by producing inflammatory cytokines, such as IFN-γ [35]. Other studies reported these cells to be anti-inflammatory [36]. During flow cytometric analysis, we gated and analysed from spleen the CD3^+^ T cells that did not express CD4, CD8, γδTCR and NK1.1 as DN T cells (S4 Fig). The frequency and numbers of DN T cells in the spleen increased dramatically, more than threefold, 1 week after sepsis induction. This increase was even greater in IL-7-treated septic mice (Fig 3A and 3B). To examine the effector functions of the DN T cell population in septic and post-septic mice, we analysed their production of IFN-γ and IL-10. One week after sepsis induction, the frequency of IFN-γ producers among the DN T cells was similar in all three groups of mice (Fig 3C). Given the highly increased absolute numbers of DN T cells in the IL-7-treated septic mice, the number of IFN-γ^+^ DN T cells was significantly increased in these mice compared with sham mice (Fig 3C). The frequency of IFN-γ producers among DN T cells continued to increase in septic mice, most prominently in the IL-7-treated septic mice throughout the observation period (Fig 3C). IL-7 treatment also increased the IL-10^+^ DN T cells shortly after administration (Fig 3E). After sepsis, the frequency and numbers of IFN-γ-expressing DN T cells consistently remained higher than IL-10^+^ cells.

### Sepsis induces a massive and long-lasting increase in MDSCs that is augmented by IL-7 treatment

**Fig 4:**
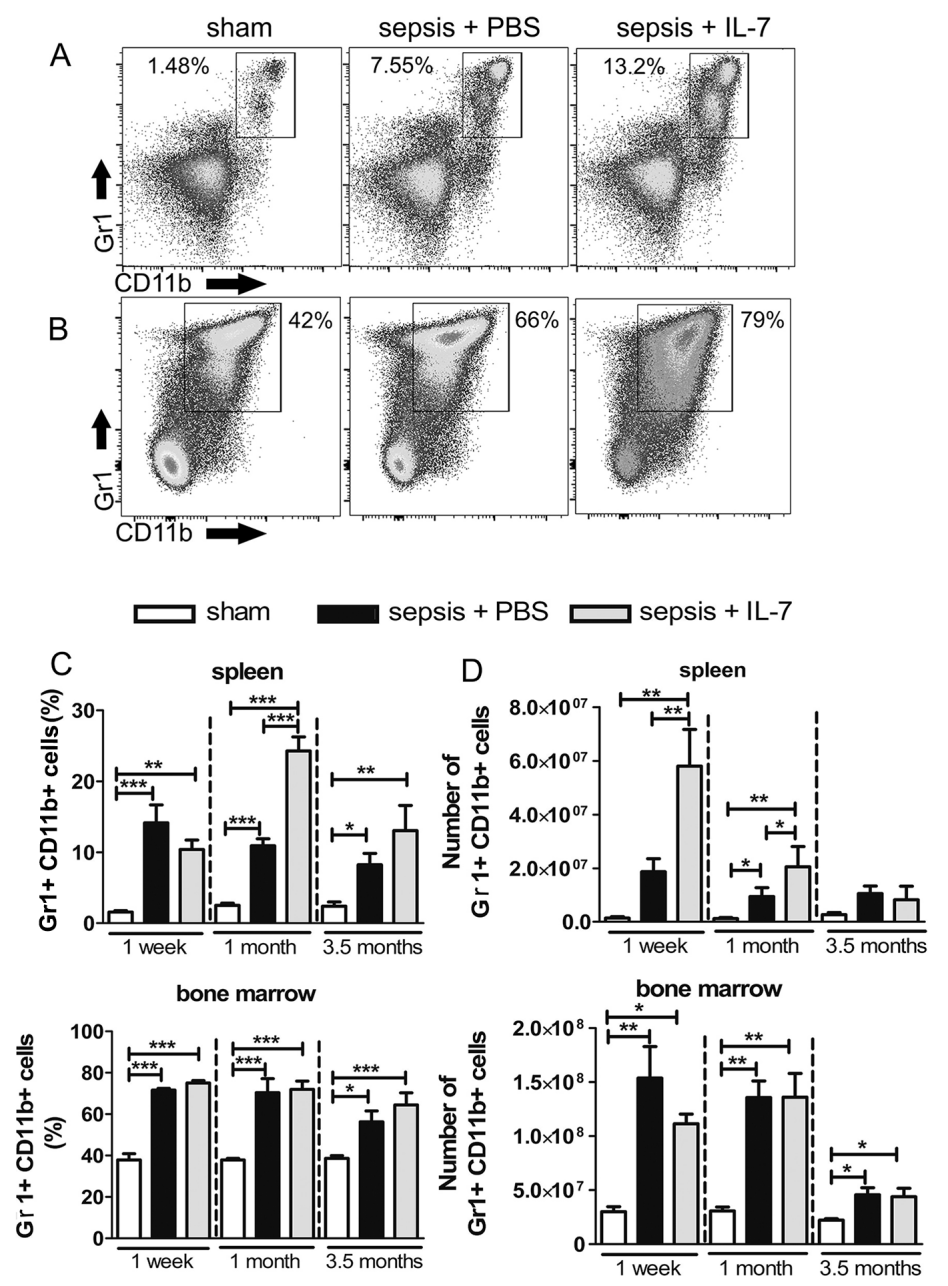
Sepsis results in a sustained expansion of MDSCs. Mice were injected with PBS i.p. (Sham) or subjected to sepsis induction. IL-7 (Sepsis + IL-7) or PBS (Sepsis + PBS) was injected daily for 5 days from day 5–9 post sepsis induction. Gr1^+^CD11b^+^ cells from the spleen and bone marrow were analysed 1 week, 1 month and 3.5 months after sepsis induction. **(A, B)** Representative flow cytometry images from spleen (A) and bone marrow (B) from analysis after 3.5 months. **(C)** Frequency of Gr1^+^CD11b^+^ cells among total spleen cells (top) and among total bone marrow cells (bottom). **(D)** Number of Gr1^+^CD11b^+^ cells in spleen (top) and bone marrow (bottom). n = 6–9 (1 week), 5–12 (1 month), 8–20 (3.5 months). *P< 0.05, **P< 0.01, ***P< 0.001 (ANOVA). Data are expressed as mean ± SEM. Data are representative of three experiments.

MDSCs are a heterogeneous group of immature myeloid cells, which dampen immune responses [17,37]. We stained spleen and bone marrow preparations for the classical markers of MDSCs: Gr1 and CD11b (Fig 4A and 4B). The frequency and numbers of Gr1^+^CD11b^+^ cells were massively increased in the bone marrow and spleen of mice with sepsis, most prominently in the septic mice treated with IL-7, compared with sham mice (Fig 4C and 4D). The frequency and numbers of MDSCs in the spleen were already increased (approximately 10 fold) in both groups of septic mice 1 week after sepsis induction compared with control mice (Fig 4C and 4D). This increase was long-lasting and even at 3.5 months after sepsis induction, the frequency of Gr1^+^CD11b^+^ cells was significantly increased in both groups of septic mice compared with controls. Remarkably, similar to our observations of DN T cells and IL-10 producing B cells, the sepsis-induced increase in this immunosuppressive cell population was further enhanced in IL-7-treated septic mice (Fig 4). The frequency of Gr1^+^CD11b^+^ cells was also significantly increased in septic mice as well as IL-7-treated septic mice in bone marrow at all of the analysed time points. Interestingly, even at the very late time point of 3.5 months after sepsis induction, the frequency of the Gr1^+^CD11b^+^ population in the bone marrow of septic and IL-7-treated septic mice remained higher than the sham mice (Fig 4C and 4D).

### Increased suppressive capacity of MDSCs from septic mice

**Fig 5:**
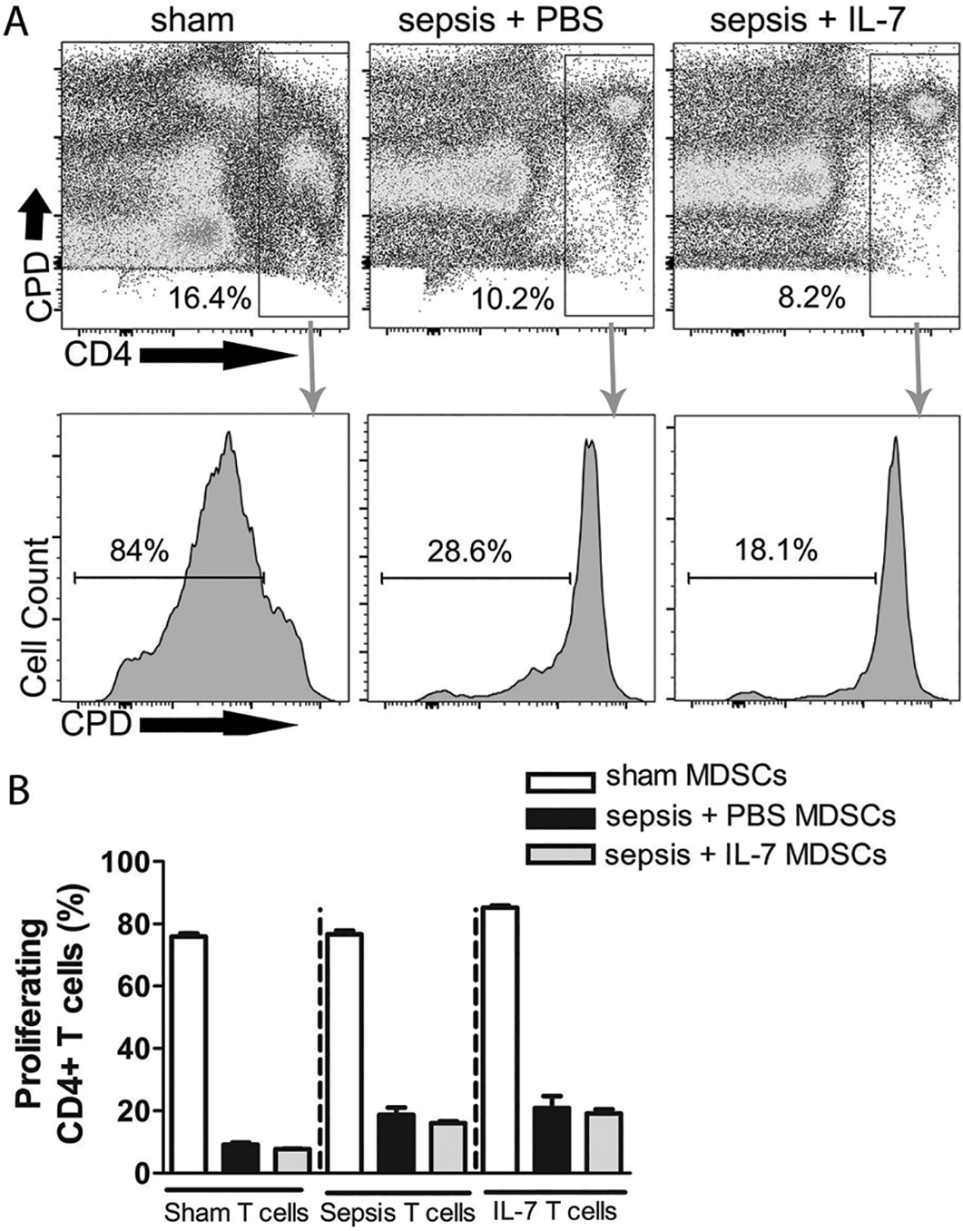
MDSCs from septic mice efficiently suppress T cell proliferation. Mice were injected with PBS i.p. (Sham) or subjected to sepsis induction. IL-7 (Sepsis + IL-7) or PBS (Sepsis + PBS) was injected daily for 5 days from day 5–9 post sepsis induction. The proliferation of CD4^+^ T cells in the presence of Gr1^+^ cells from spleen was analysed 1 week, 1 month and 3.5 months after sepsis induction. **(A)** Representative flow cytometry images from analysis after 1 month showing proliferation of T cells (determined by dilution of cell proliferation dye, CPD) from septic mice treated with IL-7 when cultured with MDSCs from sham mice (left), sepsis + PBS mice (middle) and sepsis + IL-7 mice (right). **(B)** Graph representing frequency of proliferating CD4^+^ T cells from spleen when cultured with MDSCs from spleen from different groups of mice for 3 days. This graph is representative of the experiment performed 1 month post-sepsis induction. n = 3–4 (for all time points). *P< 0.05, **P< 0.01, ***P< 0.001 (ANOVA). Data are expressed as mean ± SEM. Data are representative of one of three experiments.

The phenotypic characterization of Gr1^+^CD11b^+^ cells is a convenient starting point for identifying MDSCs, but their immunosuppressive capacity must also be demonstrated. Inhibition of T-cell activation is currently considered the gold standard to identify Gr1^+^CD11b^+^ cells as MDSCs [37]. To determine if the Gr1^+^CD11b^+^ cells possess the immunosuppressive effector functions of MDSCs, we co-cultured Gr1^+^ cells with CD4^+^ T-helper (Th) cells and analysed T cell proliferation. In this assay, Gr1^+^ cells from sham, septic or IL-7-treated septic mice were co-cultured with equal numbers of CD4^+^ T cells for 3 days. To rule out any Th-cell intrinsic difference in susceptibility to MDSC-mediated suppression, we cultured MDSCs from spleens of sham, septic or IL-7-treated septic mice with splenic Th cells from all 3 different groups of mice (Fig 5B). Approximately 80% of CD4^+^ T cells from all of the 3 groups proliferated in the presence of Gr1^+^cells from sham mice (Fig 5), similar to the proliferation observed in T cells cultured in the absence of Gr1^+^ cells (S5 Fig). In contrast, the T cell proliferation was significantly reduced upon co-culture with Gr1^+^ cells from untreated septic mice as well as IL-7-treated septic mice (Fig 5A and 5B). This finding confirmed that the Gr1^+^CD11b^+^ cells observed in septic mice are indeed immunosuppressive MDSCs. Interestingly, the T cells from IL-7-treated septic mice were slightly, if not significantly, more resistant to the effect of MDSCs compared with non-septic sham mice (Fig 5B).

## Discussion

Immunoregulatory cells have been shown in various infection models to cause immunosuppression and failure to control the infection [38–41]. This study has demonstrated massive and long-lasting expansions of IL-10-producing B cells and MDSCs in septic mice; a transient increase in number and activation of DN T cells and a very brief increase in Tregs. Three main conclusions emerge from this study. First, there is no *restitutio ad integrum* in the immune system in mice having survived acute sepsis. Secondly, our experimental study, in which we used wild-type animals, rules out a pre-existing immunodeficiency and affirms that long-lasting immunological alterations are a consequence of sepsis. In our experimental setting (*specific pathogen free* mice) secondary infections did not occur, which is clearly different from the clinical situation. It is outside the scope of this experimental study to evaluate possible clinical consequences of the prolonged expansion of immunoregulatory cells. Thirdly, the administration of IL-7 at a relatively late time point (days 5–9 post sepsis induction) did not have the same beneficial effect as previously described with earlier initiation of IL-7 treatment (starting 90 minutes after cecal ligation and puncture [24]). Instead late IL-7 treatment further increased and prolonged the expansion of immunoregulatory cell populations. Given the well-documented critical function of IL-7 for lymphocyte homeostasis, our findings do not indicate any sepsis-specific effect of IL-7 but rather reflect the physiological functions of IL-7 which also occur in the setting of sepsis (22).

IL-10-expressing B cells have been implicated in various diseases, including infections [38,39]. These cells have not been studied in sepsis. In our study, we examined B-cells for their ability to express the immunosuppressive cytokine, IL-10. Additionally, we analyzed these cells for the expression of previously defined murine phenotypic markers for so-called Bregs (CD1d^hi^CD5^+^). We show, for the first time, significantly increased frequency and numbers of IL-10 expressing B cellsin septic mice. Interestingly, the expanded IL-10^+^ B cell population remained for almost one month after sepsis induction and is, thus, likely to contribute to long-term post-sepsis immunosuppression. Moreover, IL-10^+^ B cell frequency and numbers were further increased by IL-7 treatment. Whether these cells protect against sepsis-associated hyperinflammation and/or contribute to immunosuppression causing further susceptibility to secondary infections is a topic of further investigation.

Clinical studies have revealed an increased frequency of Tregs within the diminished T cell population during the first few days after sepsis diagnosis. These findings indicate a preferential survival of Tregs compared with effector T cells in early sepsis [14,33,42] Experimental studies in preclinical models also revealed an early, transient increase in Treg frequency [14,42]. To date, it is not clear whether an increased frequency of Tregs in early sepsis is beneficial, due to their capacity to dampen hyper-inflammatory responses; detrimental, by contributing to an increased susceptibility for secondary infections; or inconsequential. The available experimental data are contradictory: Treg depletion has been reported to improve, worsen or to be inconsequential for sepsis-outcome [14,31,42]. Although Treg frequency and numbers have been studied extensively in human and experimental sepsis, there is a lack of knowledge on Tregs in sepsis-survivors. We report that there are no significant differences in Treg frequency or numbers between septic and control mice between 1 week and 3.5 months after sepsis. Interestingly, however, 1 week post sepsis, both the relative frequency and the absolute number of Tregs were significantly higher in IL-7-treated septic mice than in untreated septic mice or control mice, indicating a preferential survival of Tregs over effector T cells upon IL-7 administration. This finding confirms and extends earlier reports on IL-7-dependent survival of Tregs [43] and is, therefore, easily compatible with earlier reports on increased Treg frequency in early sepsis [14,33,42].

CD3^+^CD4^-^CD8^-^ T cells with αβTCR (DN T cells) are thought to arise after escaping negative selection in the thymus and proliferating in the periphery [20]. We report here for the first time, the increased presence of DN T cells in sepsis, particularly 1 week post sepsis. The high frequency of DN T cells even in non-treated septic mice indicates that the increase in this cell population was not primarily due to IL-7-related T cell survival and proliferation [44].

Protective as well as pathogenic effector functions have been ascribed to MDSC in sepsis [4]. We observed an expansion of MDSCs for as long as 3.5 months after sepsis induction. This confirms and extends an earlier report on lasting MDSCs expansion after sepsis [45]. Moreover, here we show for the first time, the induction of MDSCs upon IL-7 treatment, which led to an even greater increase in these cells, especially in the spleen. Whether IL-7 directly supports the development of Gr1^+^CD11b^+^ cells or whether it has an indirect effect via T cells, needs to be further investigated. The role of MDSCs in sepsis remains enigmatic. Whereas a preclinical study found that adoptive transfer of MDSCs reduced mortality in a mouse model of sepsis [46], a recent clinical study reported that an increase in MDSCs beyond the acute phase of sepsis, up to day 28, was associated with increased nosocomial infections [47]. Importantly, the MDSCs from septic mice were much more potent inhibitors of T-cell proliferation than the MDSCs from healthy controls. Given that MDSCs from non-treated and IL-7-treated septic mice strongly inhibited T-cell proliferation, the sustained presence of large numbers of these cells for months after sepsis strongly suggests that the MDSCs contribute to the lasting immunosuppression in sepsis survivors. The relative contribution of MDSC or the other immunoregulatory cell types investigated here, alone or in combination, to the immunosuppression observed in sepsis patients remains to be investigated.

## Conclusion

Our study sheds light on the expansion and function of immunosuppressive cells for up to 3.5 months after sepsis. Such a detailed long-term follow up study to analyse all the regulatory cell types has not been performed in mouse sepsis models or human sepsis patients to date. The increased and persistent presence of these cells and their immunosuppressive capacity in the PCI sepsis mouse model suggests that analysing these cells in sepsis patients and survivors is also relevant. Moreover, the fact that the frequencies of all of the investigated immunosuppressive cell populations, particularly the IL-10 producing B cells and MDSCs, were further increased by late IL-7 treatment should be considered for clinical studies.

## Supporting information

S1 Fig Survival of mice following sepsis induction and IL-7 treatment. Mice were injected with PBS i.p. (Sham) or subjected to sepsis induction. IL-7 (Sepsis + IL-7) or PBS (Sepsis + PBS) was injected daily for 5 days from day 5–9 post sepsis induction. Mice were checked for survival every day. **(A)** Experimental setup scheme. **(B)** Graph representing percentage survival of mice over the entire period of observation.
(TIF)

S2 Fig B cell population long-term post sepsis. Mice were injected with PBS i.p. (Sham) or subjected to sepsis induction. IL-7 (Sepsis + IL-7) or PBS (Sepsis + PBS) was injected daily for 5 days from day 5–9 post sepsis induction. CD19^+^ from the spleen were analyzed 1 month and 3.5 months later. **(A)** Representative flow cytometry plots from 1 month post sepsis induction. **(B)** Graphs showing number of B cells in spleen 1 month and 3.5 months post sepsis induction. n = 6. *P< 0.05, **P< 0.01 (ANOVA). Data are expressed as mean ± SEM. Data are representative of two experiments.
(TIF)

S3 Fig IL-7 treatment does not increase CD4^+^ cell numbers. Mice were injected with PBS i.p. (Sham) or subjected to sepsis induction. IL-7 (Sepsis + IL-7) or PBS (Sepsis + PBS) was injected daily for 5 days from day 5–9 post sepsis induction. The graph represents the absolute numbers of CD4^+^ T cells in the spleen, 1 week, 1 month and 3.5 months following sepsis induction. **(A)** Representative flow cytometry plots from 1 week post sepsis induction. **(B)** Graphs showing number of CD4^+^ T cells in spleen 1 week, 1 month and 3.5 months post sepsis induction. n = 6. *P< 0.05 (ANOVA). Data are expressed as mean ± SEM. Data are representative of two experiments.
(TIF)

S4 Fig Schematic showing analysis of DN T cells using flow cytometry. (TIF)

S5 Fig Controls in MDSC assay. **(A)** Representative flow cytometry images showing CD4^+^ T cell proliferation when T cells were cultured without any stimulation (negative control) and when T cells were stimulated alone without addition of Gr1^+^ cells (positive control). **(B)** Graph representing the same.
(TIF)

## Abbreviations

DN: Double negative
IL-7: Interleukin 7
IFN-γ: Interferon gamma
MDSCs: Myeloid derived suppressor cells
PCI: Peritoneal contamination and infection
Th: T-helper
Tregs: Regulatory T cells
