# Supplementary figures and images for: IL-7 treatment augments and prolongs sepsis-induced expansion of IL-10-producing B lymphocytes and myeloid-derived suppressor cells
Source: PLoS One. 2018 Feb 21;13(2):e0192304. doi: 10.1371/journal.pone.0192304 (PMC5821326; doi:10.1371/journal.pone.0192304)

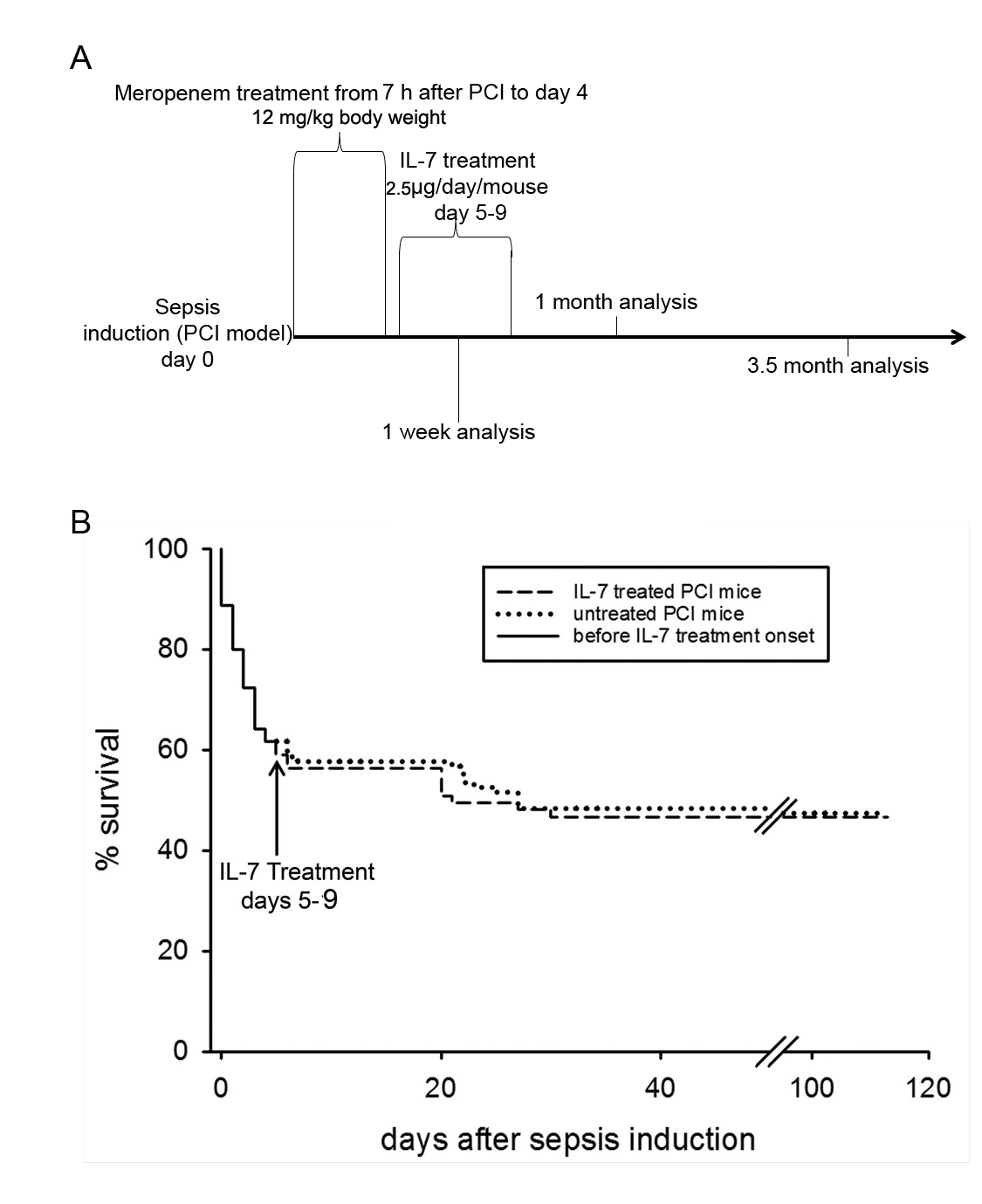

Supplement: S1 Fig — Mice were injected with PBS i.p. (Sham) or subjected to sepsis induction. IL-7 (Sepsis + IL-7) or PBS (Sepsis + PBS) was injected daily for 5 days from day 5–9 post sepsis induction. Mice were checked for survival every day. (A) Experimental setup scheme. (B) Graph representing percentage survival of mice over the entire period of observation. (TIF) [file pone.0192304.s001.tif]

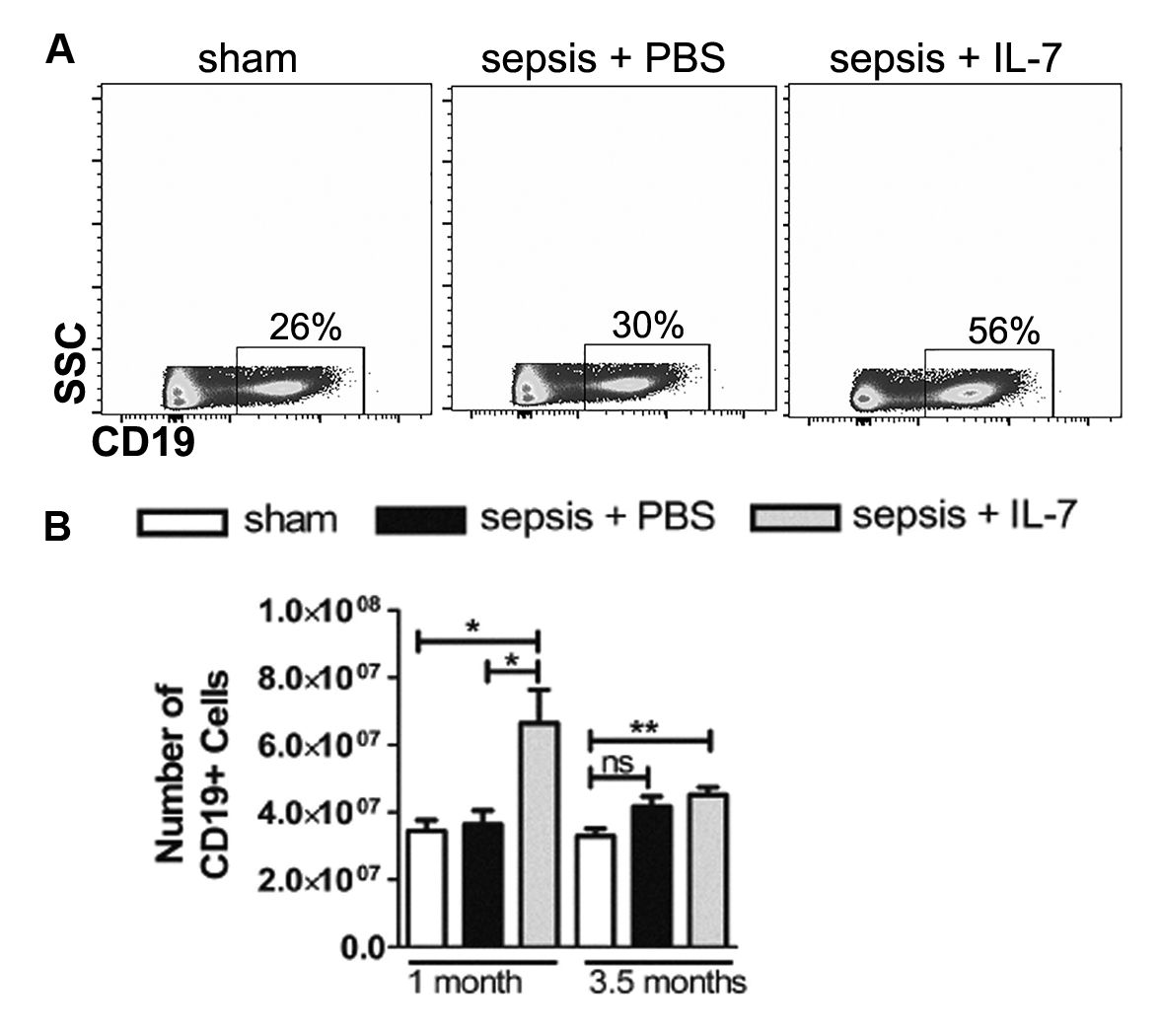

Supplement: S2 Fig — Mice were injected with PBS i.p. (Sham) or subjected to sepsis induction. IL-7 (Sepsis + IL-7) or PBS (Sepsis + PBS) was injected daily for 5 days from day 5–9 post sepsis induction. CD19+ from the spleen were analyzed 1 month and 3.5 months later. (A) Representative flow cytometry plots from 1 month post sepsis induction. (B) Graphs showing number of B cells in spleen 1 month and 3.5 months post sepsis induction. n = 6. *P< 0.05, **P< 0.01 (ANOVA). Data are expressed as mean ± SEM. Data are representative of two experiments. (TIF) [file pone.0192304.s002.tif]

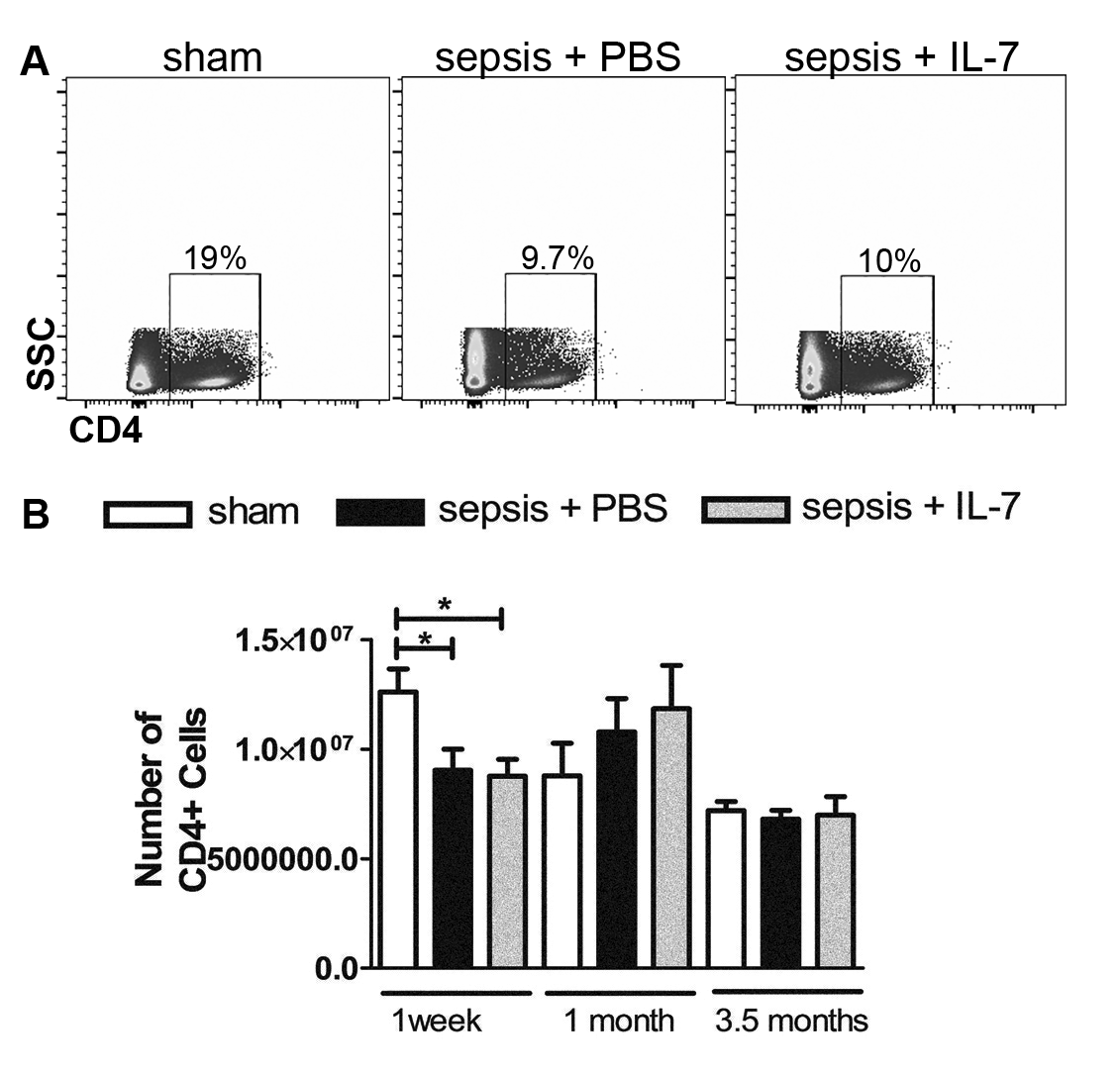

Supplement: S3 Fig — Mice were injected with PBS i.p. (Sham) or subjected to sepsis induction. IL-7 (Sepsis + IL-7) or PBS (Sepsis + PBS) was injected daily for 5 days from day 5–9 post sepsis induction. The graph represents the absolute numbers of CD4+ T cells in the spleen, 1 week, 1 month and 3.5 months following sepsis induction. (A) Representative flow cytometry plots from 1 week post sepsis induction. (B) Graphs showing number of CD4+ T cells in spleen 1 week, 1 month and 3.5 months post sepsis induction. n = 6. *P< 0.05 (ANOVA). Data are expressed as mean ± SEM. Data are representative of two experiments. (TIF) [file pone.0192304.s003.tif]

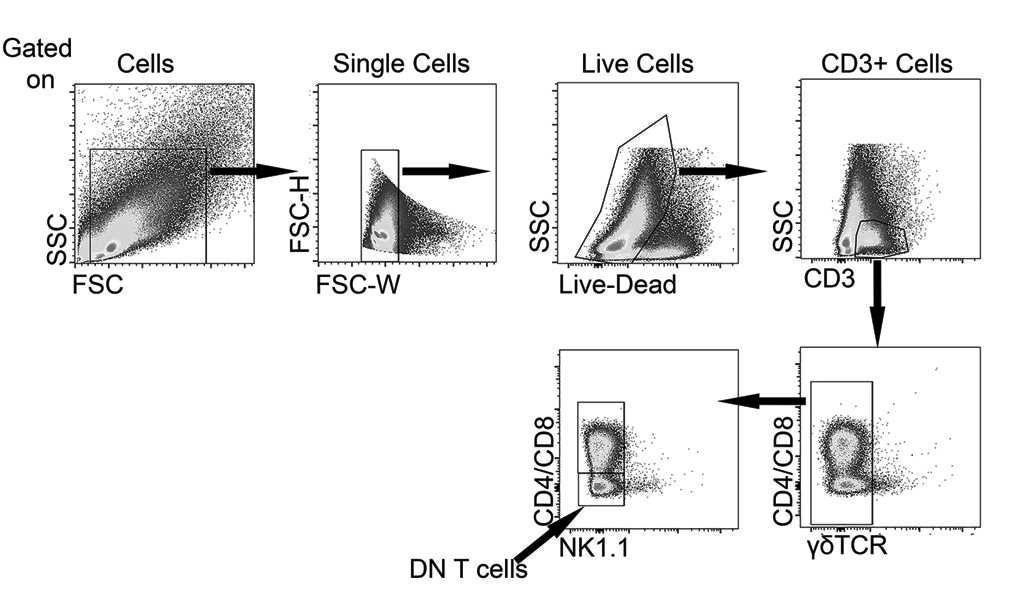

Supplement: S4 Fig — (TIF) [file pone.0192304.s004.tif]

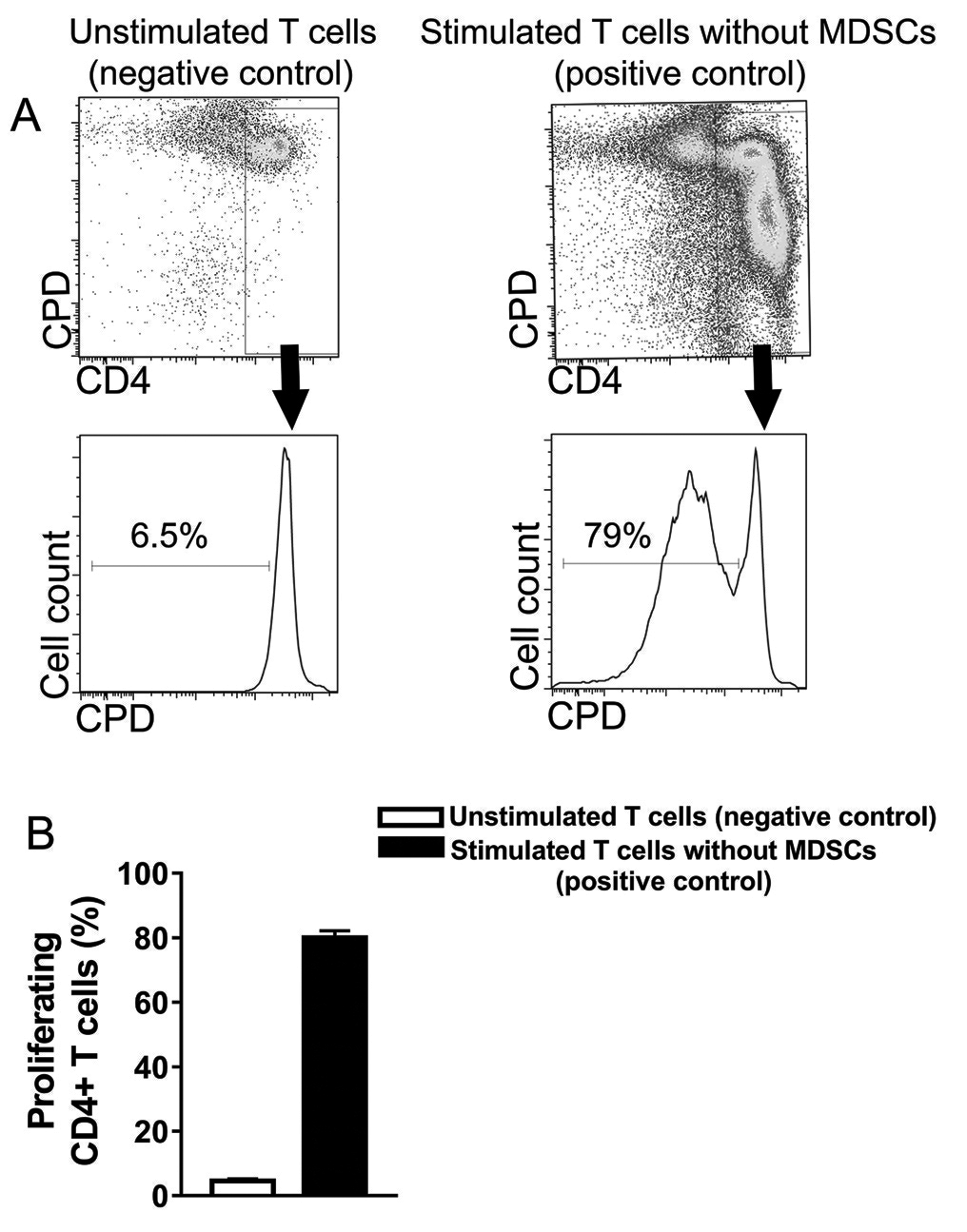

Supplement: S5 Fig — (A) Representative flow cytometry images showing CD4+ T cell proliferation when T cells were cultured without any stimulation (negative control) and when T cells were stimulated alone without addition of Gr1+ cells (positive control). (B) Graph representing the same. (TIF) [file pone.0192304.s005.tif]
